# RapidParc: A global-context transformer for parallel, accurate, and lesion-robust tractogram parcellation

**DOI:** 10.1162/IMAG.a.1168

**Published:** 2026-03-20

**Authors:** Justus Bisten, Valentin von Bornhaupt, Johannes Grün, Tobias Bauer, Theodor Rüber, Thomas Schultz

**Affiliations:** Institute for Computer Science, University of Bonn, Bonn, Germany; Department of Neuroradiology, University Hospital Bonn, Bonn, Germany; Bonn-Aachen International Center for Information Technology (b-it), Bonn, Germany; Department of Epileptology, University Hospital Bonn, Bonn, Germany; German Center for Neurodegenerative Diseases (DZNE), Bonn, Germany; Center for Medical Data Usability and Translation (ZMDT), Bonn, Germany; Lamarr Institute for Machine Learning and Artificial Intelligence, Bonn, Germany

**Keywords:** diffusion MRI, white matter bundles, streamline classification, tractography segmentation, hemispherotomy, and lesion robustness

## Abstract

Whole-brain diffusion MRI tractography produces tractograms, dense sets of streamlines that represent white matter architecture. Structural connectivity studies and clinical pipelines often involve tractogram parcellation, a process in which each streamline is assigned to an anatomical bundle or identified as a false positive. Recent advances made tractogram parcellation registration-free, considering the computational effort of registration, and the challenges posed by the registration of pathological cases. We introduce RapidParc, a novel transformer-based method for registration-free tractogram parcellation. RapidParc treats each streamline as a token and processes many of them in parallel. This way, they serve as a global context for each other, permitting accurate classification while the high level of parallelism leads to rapid computation. Our design is two orders of magnitude faster than TractCloud, a recent state-of-the-art method for registration-free parcellation, and supports CPU-only inference, while achieving even slightly higher accuracy. Comparing RapidParc to TractCloud in a cohort of 22 individuals post-hemispherotomy and 30 individuals after selective amygdalohippocampectomy (sAH), it generalizes better to structurally altered anatomy, even when trained exclusively on data from healthy controls. This intrinsic robustness is further improved by applying a novel augmentation strategy during training. Finally, we investigate the main factors that contribute to that improved generalization. Our results highlight the importance of robust tractogram centering in registration-free approaches. They also suggest that constructing a local context for each streamline, on which TractCloud spends considerable computational resources, does not appear to contribute to its accuracy.

## 1 Introduction

Diffusion magnetic resonance imaging (dMRI) tractography (Jeurissen et al., 2019) is unique in its ability to visualize and quantify major white matter tracts within the brain in vivo, by leveraging the self-diffusion of water molecules (Basser et al., 1994). It is widely used in both clinical and research settings (Azeem et al., 2024; Catani et al., 2013; Gadot et al., 2023; Jeong et al., 2024; Lan et al., 2025; Petit et al., 2022; Warrington et al., 2020; Zhang et al., 2021). Clinically, tractography supports neurosurgical planning by mapping functionally critical white matter tracts adjacent to lesions or resection paths, thereby minimizing the risk of postoperative deficits (Chamberland et al., 2014; Coenen & Reisert, 2022; Gurses et al., 2025; Kamagata et al., 2024; Tax et al., 2012, 2014). In research, it enables analysis of white matter microstructure and large-scale structural connectivity, uncovering abnormalities even outside lesion sites (Bisten et al., 2025; Maallo et al., 2019).

However, whole-brain tractography generates dense tractograms for comprehensive white matter mapping (Rheault et al., 2017; Yang et al., 2021) which require post-processing. Tractogram parcellation involves eliminating false positives and assigning streamlines to anatomical bundles (Zhang, Wu, Norton, et al., 2018, Zhang et al., 2020). While major white matter bundles can be manually delineated, such parcellation relies on the presence of anatomical landmarks (Barbeau et al., 2020; Catani et al., 2005; Forkel et al., 2025), is laborious, inconsistant, and prone to annotation bias (Colon-Perez et al., 2016; Karimi & Warfield, 2024; Rheault et al., 2020; Schilling et al., 2021; Zhang, Wu, Norton, et al., 2018). Thus, automating this process has been a central research focus (Bertò et al., 2020; Chen et al., 2023; Kumaralingam et al., 2022; Ngattai Lam et al., 2018; Xue et al., 2023; Zhang, Wu, Norton, et al., 2018, Zhang et al., 2020), including connectivity-based models that classify streamlines by intersection with predefined anatomical regions (Wassermann et al., 2016), and streamline-based models that assign tracts based on learned or geometric similarity to reference bundles (Bertò et al., 2020; Garyfallidis et al., 2018). These approaches address several issues that arise when processing dMRI streamlines, such as their lack of orientation, implying that representations in forward or reversed order should be treated equivalently.

Early methods relied on spatial registration into template spaces (Bertò et al., 2020; Garyfallidis et al., 2018; Wassermann et al., 2016; Zhang et al., 2020). However, anatomical deformations in lesioned brains can compromise registration accuracy (Ripollés et al., 2012) and make registration-based methods falter. Tractography is disrupted in pathological contexts such as ischemic stroke, tumors, traumatic brain injury, and demyelinating diseases (Lazar & Alexander, 2006; Yeh et al., 2021). Lesions can disrupt or displace tracts, introduce peritumoral edema, and infiltrate white matter, all of which distort diffusion signals (Chen et al., 2015; Min et al., 2013; Wang et al., 2011; Yeh et al., 2021). However, accurate delineation of tracts in such conditions is critical for guiding resective surgery or quantifying the loss of connectivity post-injury (Caeyenberghs et al., 2013; Wilde et al., 2006). In cases of severe pathology, such as Rasmussen’s encephalitis or in individuals post-hemispherotomy, a last-resort operation for drug-resistant epilepsy (Delalande, 1992; Delalande et al., 2007), standard methods often produce implausible reconstructions or fail to classify tracts altogether (Bauer et al., 2024; Gruen et al., 2025; Held et al., 2023).

Considering such issues as well as the computational effort of registration, Xue et al. (2023) introduced TractCloud as a registration-free model that classifies each streamline independently using a local-global point cloud architecture. TractCloud is faster than registration-based alternatives, and achieves even higher accuracy, but the construction of an individual local-global context for each streamline is still relatively costly.

Our first contribution is to introduce RapidParc as a transformer-based alternative to TractCloud. Transformer architectures (Vaswani et al., 2017) have recently seen promising adoptions in tractography analysis tasks. For instance, TractGraphFormer integrates anatomically informed graph convolutions with self-attention to learn global relationships and predict sex and age from diffusion-derived fiber-cluster features (Chen, Zhang, et al., 2025). RapidParc leverages the self-attention mechanism (Vaswani et al., 2017) to exchange information among streamlines, building up a feature space representation that is suitable for registration-free classification. This way, streamlines serve as a global context for each other, while being processed in parallel, which enables rapid computation and powerful data augmentation. We demonstrate that, despite the lack of a dedicated local context, our architecture achieves slightly higher accuracy than TractCloud. At the same time, it is two orders of magnitude faster. This is particularly important in large-scale neuroimaging studies, where millions of streamlines must be processed, as well as in clinical settings, where modern GPU resources are not always available. Therefore, we also demonstrate that our approach is efficient enough to make inference feasible in a CPU-only setting.

Our second contribution is to evaluate the robustness of registration-free tractogram parcellation to severe white matter disruptions, and to investigate factors contributing to it, using a dataset comprising 22 individuals who underwent hemispherotomy at the University Hospital Bonn. We find that RapidParc generalizes better to those cases than TractCloud, even when trained only on healthy subjects, and that this can be improved further via additional data augmentation. Investigating how specific differences between TractCloud and RapidParc affect generalization suggests that the exact way in which tractograms are centered matters in cases of asymmetric white matter loss. On the other hand, the use of a local context in TractCloud does not appear to have a major effect on its accuracy.

## 2 Materials and Methods

**Fig. 1.**
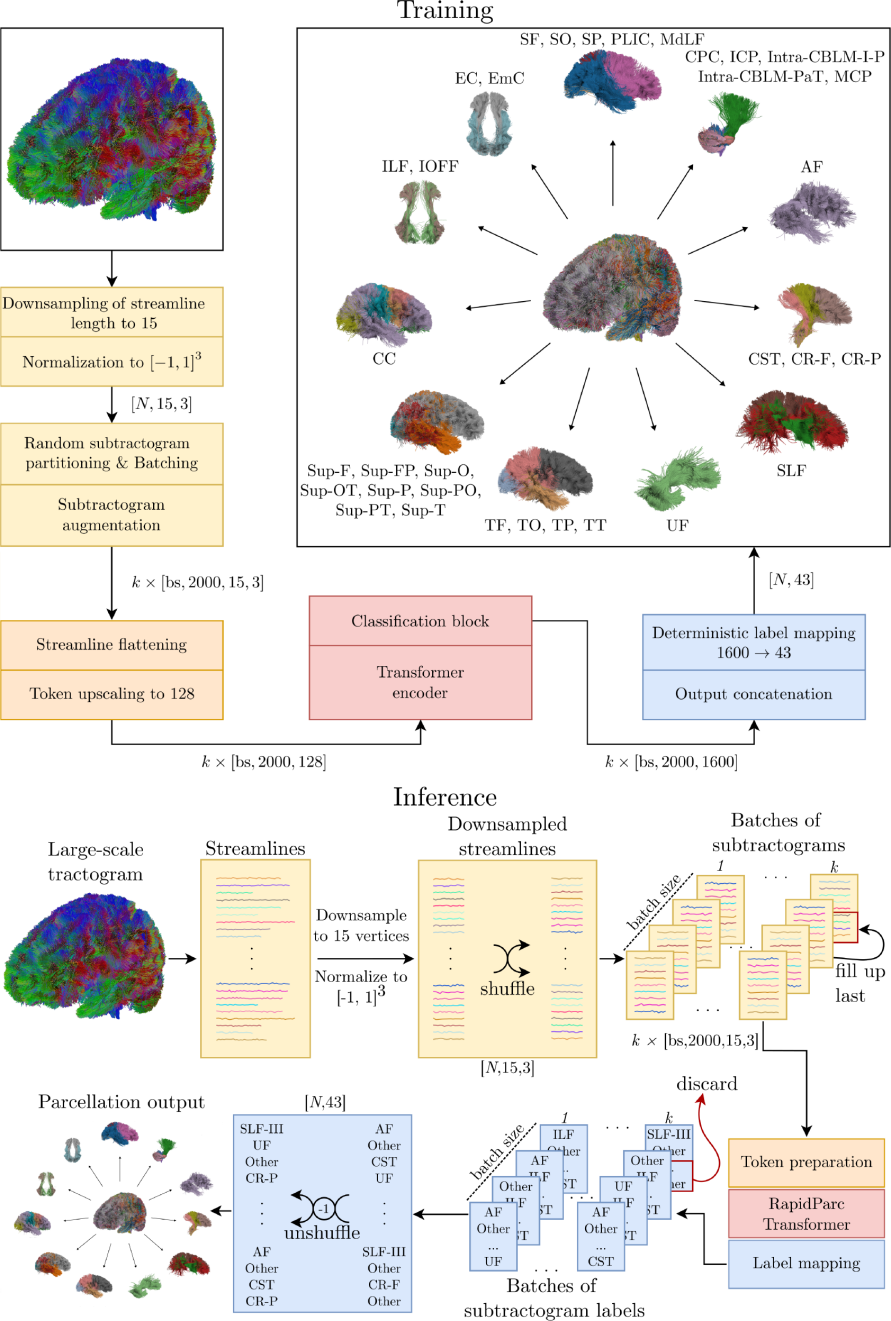
Our pipeline consists of the four stages preprocessing (yellow), embedding/token preparation (orange), transformer encoder/classifier (red), and postprocessing (blue). A full list of tract names and abbreviations is available in the ORG atlas of the *whitematteranalysis* package (O’Donnell et al., 2007; Zhang, Wu, Norton, et al., 2018).

In this work, we propose RapidParc, a transformer-based model for fast, highly-parallelized tractography parcellation that fully operates on global tractogram context and does not rely on image registration. By omitting computation of local streamline neighborhood embeddings, RapidParc enables highly parallelized inference while maintaining state-of-the-art accuracy across diverse and lesioned populations. An overview over the RapidParc pipeline is shown in Figure 1.

### 2.1 Data

For training and quantitative evaluation, we use the dataset and labels provided by TractCloud (Xue et al., 2023). This dataset defines 1600 clusters which are mapped to 42 bundles and one outlier class for evaluation. For external validation, we directly compare results from RapidParc to TractCloud on tractograms from four independently acquired subjects from the developing Human Connectome Project (dHCP) (Edwards and et al., 2022), the Adolescent Brain Cognitive Development (ABCD) study (Casey et al., 2018; Volkow et al., 2018), the HCP (Van Essen et al., 2012), and the Parkinson’s Progression Markers Initiative (PPMI) (Marek et al., 2018). To compare performance on tractograms in severely lesioned brains, we compare our model to TractCloud on a dataset containing individuals post-hemispherotomy.

#### 2.1.1 Data post-hemispherotomy and selective amygdalohippocampectomy

**Fig. 2.**
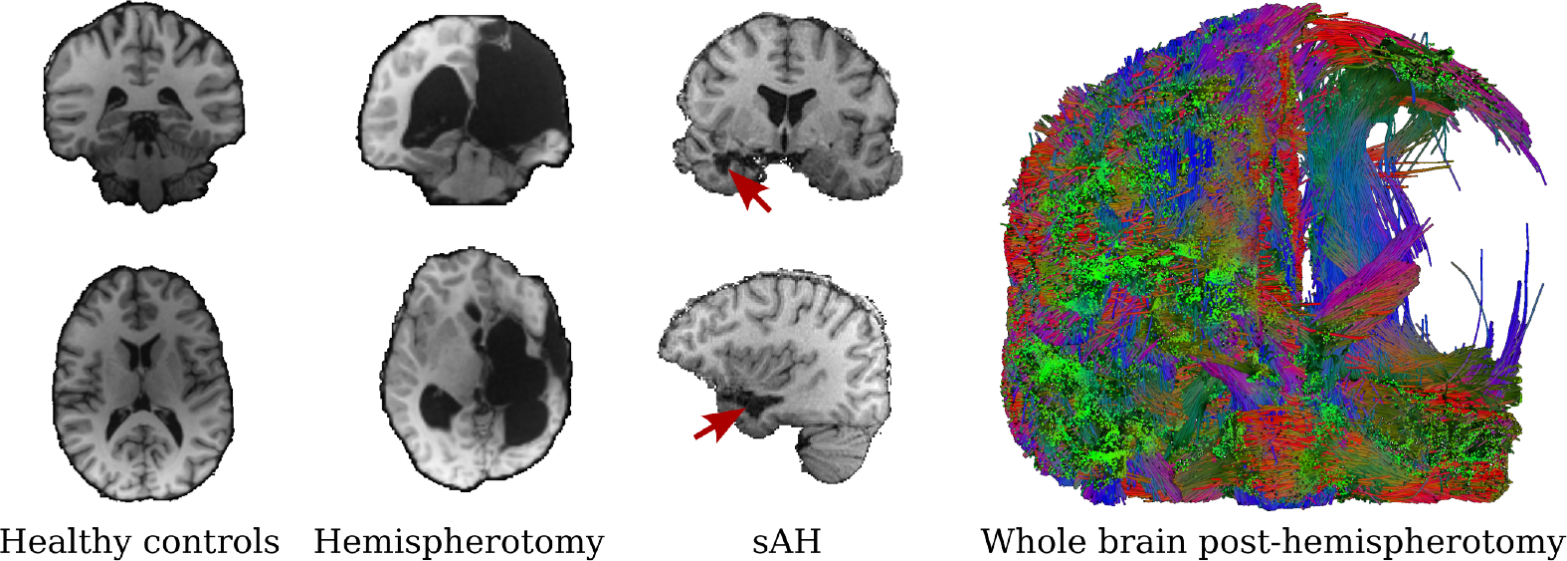
Whole-brain tractography post-hemispherotomy. Left: Representative coronal and axial T1-weighted MRI slices from a healthy control (left column), an individual post-hemispherotomy (middle column, extensive resection and loss of tissue), and an individual after sAH (right column, resection site indicated by red arrows). Right: Whole-brain tractography from an individual post-hemispherotomy with a substantial reduction of streamlines in the lesional hemisphere.

MRI data of individuals post-hemispherotomy, after selective amygdalohippocampectomy (sAH) and healthy controls used in this work were originally acquired as part of clinical and neuropsychological investigation of epilepsy surgery outcomes at the University Hospital Bonn (Bauer et al., 2024; Bisten et al., 2025; David et al., 2021). For the current study, we leverage these existing data to evaluate tractography and parcellation methods in the context of severe white matter disruptions following hemispherotomy and sAH (see Fig. 2). Subjects for the hemispherotomy dataset were retrospectively selected from individuals with epilepsy treated at the Department of Epileptology, University Hospital Bonn, who had undergone hemispherotomy for medically refractory epilepsy, as well as from healthy control subjects. Inclusion criteria were: (1) native German language proficiency, (2) absence of MRI contraindications, (3) ability to undergo approximately 2 h of MRI scanning. Data collection occurred between 2011 and 2014. The study initially enrolled 20 healthy controls and 34 individuals who had undergone hemispherotomy between 1992 and 2012. Of the individuals post-hemispherotomy, nine were excluded due to inability to complete the imaging protocol. An additional two were excluded for missing neuropsychological data, and one was excluded due to excessive image noise that precluded reliable tractography. The final sample, thus, comprised 42 participants: 22 individuals post-hemispherotomy and 20 healthy controls. Additionally, we investigated 30 individuals with pharmacoresistant mesial temporal lobe epilepsy. These subjects underwent sAH at the Department of Neurosurgery, University Hospital Bonn, between 2009 and 2012. The sAH was performed using either a subtemporal or a transsylvian approach. T1-weighted images and DWI were acquired several months after the procedure. Both studies were approved by the Institutional Review Board of the medical faculty of the University of Bonn. Informed consent was obtained from all participants and/or their legal guardians.

MRI data were acquired on a Siemens Magnetom Trio 3T scanner equipped with an 8-channel head coil. The imaging protocol included whole-brain T1-, T2-, and diffusion-weighted sequences. High-resolution T1-weighted MP-RAGE images (1.0 mm isotropic, 160 slices) and 3D-T2-weighted images (1.0 mm isotropic, 192 slices) were obtained. Diffusion-weighted imaging employed single-shot, dual-echo, spin-echo EPI with 72 axial slices ($1.72\times 1.72\times 1.7\;{\text{mm}}^{3}$), 60 diffusion directions ($b=1000\;\text{s}/{\text{mm}}^{2}$), and six $b=0\;\text{s}/{\text{mm}}^{2}$ baseline volumes. All data were converted to the Brain Imaging Data Structure (BIDS) format (Gorgolewski et al., 2016), and preprocessed using FSL 6.0.7 (Jenkinson et al., 2012). Diffusion data were denoised and underwent eddy current correction, with mean b=0 images extracted via *dwiextract*. T1-weighted images were skull-stripped using *robustfov* and *bet2*. Tissue segmentation was performed using FSL FAST (Zhang et al., 2001). Fiber orientation distribution functions (fODFs) were estimated according to methods described by Ankele et al. (2017). For the whole-brain tractography, a low-rank tensor approximation of fODFs using an unscented Kalman filter was employed, providing spatial regularization (Gruen et al., 2023; Schultz & Seidel, 2008). The tractography pipeline is available as part of the *bonndit* package (https://github.com/MedVisBonn/bonndit). Streamlines with a length below 40 mm were filtered.

We evaluate our transformer model on whole-brain tractograms of both cohorts, containing approximately 1 million streamlines each. To generate ground-truth tract labels for this dataset, we used the O’Donnell Research Group (ORG) atlas-based tractogram parcellation pipeline, provided in the *whitematteranalysis* toolkit (O’Donnell et al., 2007; Zhang, Wu, Norton, et al., 2018). To bring the tractograms of individuals’ post-hemispherotomy into the ORG atlas space, we first applied manually curated affine and nonlinear Advanced Normalization Tools (ANTs)-based transformations (Klein et al., 2009), to align each subject’s diffusion data to MNI152 space (Fonov et al., 2009). We then performed an additional registration step to align the MNI152 1 mm T1-weighted template to the ORG population-mean T1 template using ANTs. This involved rigid, affine, and SyN transformations.

The resulting composite warp was used to transform streamlines from MNI152 space into the ORG atlas space, after which streamlines were clustered using spectral embedding and matched to 1600 clusters through spectral-atlas label propagation (O’Donnell et al., 2007; Zhang, Wu, Norton, et al., 2018); half of these clusters represent atlas-defined bundles, and the other half capture false positive detections. To improve anatomical specificity, outliers were removed using a similarity-based filtering procedure with a two standard deviations threshold as proposed by O’Donnell et al. (2017); Zhang, Wu, Ning, et al. (2018). The cleaned and labeled tractograms were subsequently mapped to the 42-class tract-level parcellation provided by Zhang, Wu, Norton, et al. (2018) and used as ground-truth labels for evaluating the automated parcellation methods under conditions of severe anatomical disruption observed in the hemispherotomy data.

Our analysis compensated for the fact that scans in our datasets had a reduced inferior field of view (FOV) relative to the ORG atlas, a known source of bias for similarity-based parcellation (Chen, Zekelman, et al., 2025). To harmonize the atlas and our data, we truncated the ORG atlas reference tractography to each subject’s FOV prior to any similarity computations, including outlier filtering. We determined the subject-specific inferior FOV boundary after mapping it to atlas space and removed atlas geometry below it. Atlas streamlines intersecting the boundary were cropped; if this only preserved two or fewer vertices, similarities toward that streamline were set to zero when computing the spectral embedding. This procedure left subject tractograms unchanged, preserved atlas topology, restored consistency between atlas and observed data, and substantially improved recall for classes whose streamlines leave the FOV.

### 2.2 Data preprocessing

Streamlines were resampled to a fixed length of 15 points. For tokenization, streamlines were flattened to 45 dimensions, then padded to $d_{\text{model}}=128$ dimensions by filling the remaining entries with the output of a linear layer. Our architecture is readily extensible to support higher-resolution streamline representations, which may better preserve features such as high curvature, by appropriately adjusting the initial tokenization scheme. As the choice of 15 points did yield high accuracy and is widely adopted in the literature (Xue et al., 2023; Zhang et al., 2024), exploration of alternative, higher-resolution representations was left to future work. Coordinates were normalized to ${\left[-1,\;1\right]}^{3}$ using affine min-max normalization along each axis. This ensured that all streamlines were transformed into a common space and scale, making the representation invariant to translation and scaling and eliminating the need for axis-specific scaling augmentations. Similar normalization has been used by Hendriks et al. (2025) for implicit continuous fODF modeling.

### 2.3 Model architecture & training

The backbone of our proposed model is a transformer encoder consisting of eight layers. Each layer uses single-head self-attention and a feed-forward network with 256 hidden units. We used a token dimension of 128, for which we observed optimal model accuracy. A dropout rate (Srivastava et al., 2014) of 0.1 is applied within each layer, along with layer normalization (Ba et al., 2016). The output from the transformer encoder is passed through a 256 hidden units classification block and an ReLU activation, followed by a second linear layer with 1600 output units, corresponding to the streamline clusters.

The model was trained using the Adam optimizer (Kingma & Ba, 2014) with a learning rate of $8.5\cdot 10^{-4}$ , a weight decay of $10^{-3}$ , and a cosine annealing learning rate scheduler. Training was performed with a batch size of 64 for 10,000 epochs, employing a cross-entropy loss function (Goodfellow et al., 2016). Inverse class frequency weighting of the loss did not yield performance improvements and was therefore not employed. Training RapidParc took approximately 5 h and 15 min, using a distributed learning strategy on two NVIDIA A40 GPUs with 48 GB of memory each.

We used the same training, validation, and test split as in TractCloud to ensure direct comparability between methods (Xue et al., 2023). The 1600 clusters from the ORG atlas were used as labels during training (Zhang, Wu, Norton, et al., 2018). Each sample in a batch consisted of a random subtractogram of context_size = 2000 from a single training subject.

**Appendix Fig. A.1.**
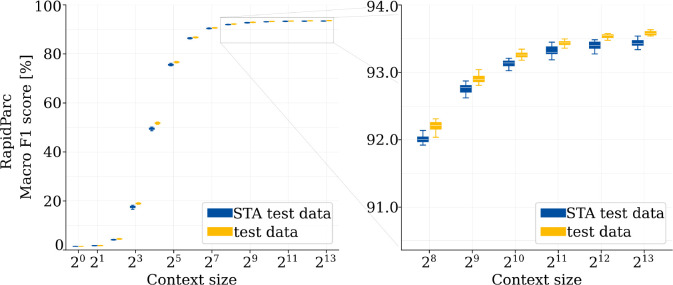
Influence of context_size on RapidParc macro F1 score (%). Plots show context size on a logarithmic scale for both standard test data (200,000 streamlines, yellow) and STA test data (6.2 million streamlines, blue). Registration-free parcellation clearly requires sufficient context, but scores saturate at a certain point. The relevant range is shown in more detail in an inset.

**Appendix Fig. A.2.**
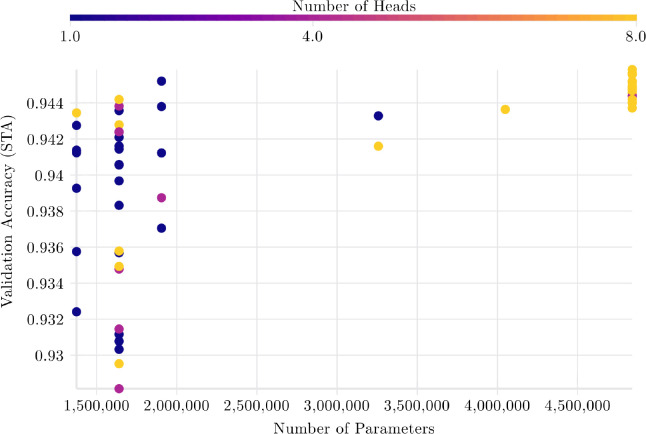
Validation accuracy over the number of model parameters for various combinations of hyperparameters. The number of attention heads is color coded. The best model with multiple heads achieves only marginally higher accuracy than the best model with a single head, but requires a substantially higher number of parameters. All evaluations used 30 STA augmentations and a fixed context_size of 2000.

**Appendix Fig. A.3.**
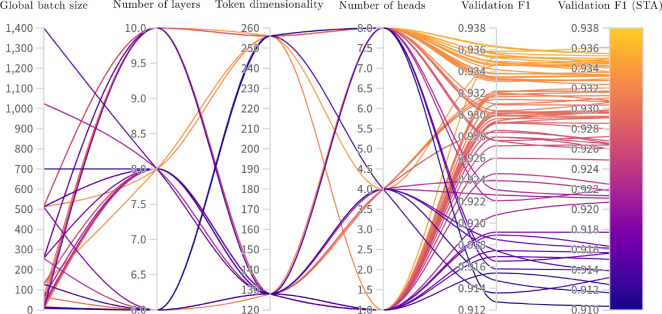
Parallel coordinate plots of the results of our hyperparameter search, including the number of heads, number of layers, token dimensionality (d_model), and F1 scores for the STA validation dataset (30 augmentations) and validation dataset without augmentations.

**Appendix Fig. A.4.**
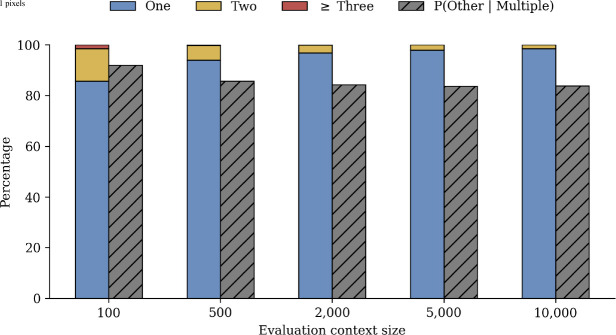
Due to the random grouping into subtractograms, streamlines might be classified differently in repeated runs. The stacked bar (blue/yellow/red) shows how frequently this happened over 20 runs, depending on the context_size. Yellow indicates how frequently streamlines were assigned to their second most frequent class, red indicates assignment to a third or even rarer class. Hatched gray bars show the fraction of inconsistently classified streamlines (“Multiple”) that are moved into the outlier class (“Other”) at least once, illustrating that inconsistency mostly affects streamlines at the boundary of being accepted as inliers.

**Appendix Fig. A.5.**
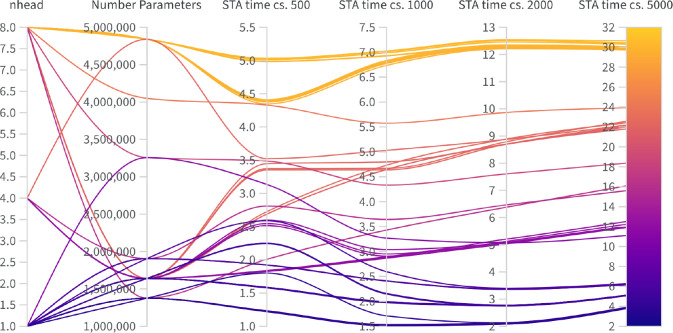
Parallel coordinate plots of the number of heads, and the inference time on the STA validation dataset. All time measurements were conducted on a single NVIDIA A40 GPU and using the PyTorch inference mode.

Hyperparameters were established by systematically varying the number of transformer blocks and attention heads per block, token dimensionality, and batch size (Appendix Fig. A.3). Larger context sizes increase accuracy (Appendix Fig. A.1) and prediction stability (Appendix Fig. A.4), but additional benefits diminish beyond context_size = 2000 while computational effort increases disproportionally due to the quadratic complexity of self-attention with respect to the number of tokens. Similarly, a larger number of attention heads increased the number of parameters (Appendix Fig. A.2) and computational effort (Appendix Fig. A.5) without a clear benefit. During evaluation, multiple subtractograms were combined into batches. Using less than 10 GB of GPU memory, we process 512 subtractograms simultaneously, containing more than one million streamlines.

Data augmentation following TractCloud (Xue et al., 2023) included rotations with angles sampled uniformly from [−45^°^,45^°^] for the left-right axis, and [−10^°^,10^°^] for both the anterior-posterior and superior-inferior axes. Since our approach does not require recalculating local or global features after augmentation, these rotations were applied in every epoch for every subtractogram, resulting in approximately 3.5 million rotation augmentations.

Additionally, Gaussian noise with σ=0.001 was added to each point of the streamlines. The streamlines were then re-normalized to ${\left[-1,\;\;1\right]}^{3}$ to ensure that the scale of the transformer input remained consistent despite the effects of rotation and noise. To promote directional invariance, we implemented a flip-augmentation step in which the order of points within each streamline was randomly reversed with probability $p_{\text{flip}}=0.5$ during training, ensuring that the augmentation is applied in an i.i.d. manner. This approach generates a high diversity of augmented streamlines within each subtractogram.

Due to the inherent permutation equivariance of the attention mechanism (Vaswani et al., 2017), the order of streamlines within a subtractogram does not affect their classification. Moreover, the fact that transformers support sequences of variable length allows us to evaluate our architecture with context sizes deviating from those used during training.

### 2.4 Hemisphere dropout as data augmentation

In a variant of our model, we experimented with a new data augmentation strategy in which a fraction $p_{\text{hemi}}=0.3$ of subtractograms used during training only contain streamlines from a single hemisphere. This should encourage the transformer to primarily rely on streamlines from the same hemisphere while building up its feature space representation. We hypothesized that this should improve generalization from healthy training data to tracts in the preserved hemisphere in individuals post-hemispherotomy, where contralateral fibers are most strongly affected, and that it might not have a great effect on healthy controls, since ipsilateral fibers might provide sufficient context for reliable classification.

We refer to the resulting model variant as ${\text{RapidParc}}_{\text{hemiaug}}$ . For building unilateral subtractograms, streamlines were assigned to one of the hemispheres based on $\bar{x}$ , their centroid x-coordinate. Streamlines with $\bar{x}<0$ were considered left hemisphere, while those with $\bar{x}\geq 0$ were assigned to the right. During augmentation, all streamlines in the subtractogram were sampled from the selected hemisphere’s pool according to this assignment. ${\text{RapidParc}}_{\text{hemiaug}}$ was trained with the same context_size of 2000 as the previously described model variant.

### 2.5 Model evaluation & statistical analysis

For model comparison, we adopted the evaluation protocol established by TractCloud (Xue et al., 2023). Overall model performance was quantified using accuracy and macro-averaged F1 score, computed at the streamline level across all anatomical bundles. Each prediction was mapped from the initial 1600 ORG atlas-cluster labels to the 42 anatomical tract classes and the outlier class. Accuracy was defined as the proportion of correctly classified streamlines. The macro-averaged F1 score was obtained by averaging the per-tract F1 scores across all 42 anatomical bundles, excluding the outlier class. To assess computational efficiency, we recorded the total model inference time, defined as the elapsed time required for feature extraction and forward propagation of the entire test set, excluding data loading.

All evaluations were performed using the same test set splits as in TractCloud, enabling direct comparison of both predictive performance and inference efficiency. For the comparison of model performance on the hemispherotomy dataset, cerebellar tracts were excluded due to the reduced field-of-view in the respective dMRI acquisitions. Furthermore, commissural tracts, including the corpus callosum, were excluded from analysis in individuals post-hemispherotomy, as these bundles are surgically disconnected. Model performance was then assessed at the streamline level for each anatomical bundle using precision, recall, and F1 score. All metrics were computed per tract and subject, then aggregated by group (control or post-hemispherotomy).

To evaluate statistical significance between models, including our RapidParc approach, TractCloud, and ablations, pairwise Wilcoxon signed-rank tests were performed for each tract (Wilcoxon, 1945), with resulting p-values corrected for multiple comparisons across all tests using Bonferroni’s method (Neyman & Pearson, 1928).

### 2.6 TractCloud ablations

To investigate the reasons for the observed differences between the results of RapidParc and TractCloud, we implemented two ablations of TractCloud. First, Xue et al. (2023) themselves compare a full version of TractCloud to an ablation that only uses local context, and find that combining local and global context achieves higher accuracy. However, they do not compare to a variant that only uses the global context. Since the latter corresponds to the strategy used in our work, we performed that missing ablation, presenting results from a variant GlobalTractCloud in which all local context computations were removed, while the original model architecture and hyperparameters were preserved.

Second, TractCloud centers input tractograms via their center of mass, while the centering in RapidParc is implicit in the coordinate normalization to ${\left[-1,\;1\right]}^{3}$. We hypothesized that the latter might be more robust in cases of incomplete or spatially asymmetric tractograms (see Fig. 2), as it does not assume an even distribution of streamlines.

Therefore, we implemented TractCloud_bb_, a variant of TractCloud that uses a bounding-box centering approach. For each subject, the tractogram center ${\text{c}}_{\text{bb}}$ was defined as the midpoint between the minimum and maximum streamline coordinates along each axis,

$$\begin{array}{cccc} {\text{c}}_{\text{bb}} & =\frac{1}{2}\left(\underset{i}{\text{min }}{\text{x}}_{i}+\underset{i}{\text{max }}{\text{x}}_{i}\right), & & \end{array}$$

and the resulting displacement relative to the ORG atlas bounding-box center ${\text{c}}_{\text{atlas}}$ , was applied to all streamline coordinates,

$$\begin{array}{cccc} {\text{x}}_{i\text{,centered}} & ={\text{x}}_{i}+\left({\text{c}}_{\text{atlas}}-{\text{c}}_{\text{bb}}\right). & & \end{array}$$

In all ablation experiments, features were computed in the same manner as in the original TractCloud, except for the specific component under investigation.

## 3 Results

### 3.1 Performance on test dataset

A quantitative comparison of accuracy and running times on the same test set that was used by Xue et al. (2023) is shown in Table 1. In both respects, RapidParc surpasses both TractCloud, the state-of-the-art registration-free tractography parcellation method (Xue et al., 2023), and its global-only ablation variant GlobalTractCloud. On the unaugmented test set (200.000 streamlines), ${\text{RapidParc}}_{\text{2000}}$ achieves 94.44% accuracy and 93.2% macro F1 score, representing absolute improvements of 2.32 (2.26) and 2.98 (3.06) percentage points over TractCloud and GlobalTractCloud, respectively. We also evaluated model performance on the synthetic transform augmented (STA) dataset comprising 6.2 million streamlines obtained by augmenting the test dataset with rotations uniformly sampled from [−45^°^,45^°^] about the left-right axis and [−10^°^,10^°^] about the anterior-posterior and superior-inferior axes, translations of [−50, 50] mm along each axis, and scaling from −45% to +5% applied along all three axes (Xue et al., 2023). ${\text{RapidParc}}_{\text{hemiaug}}$ maintains the highest performance with 93.86% accuracy and 92.36% F1, while requiring only 5.44 s on a single NVIDIA A40 GPU. In contrast, TractCloud requires 703.92 s for the same task, implying a 129 × decrease in runtime when using RapidParc. Furthermore, comparison of TractCloud and GlobalTractCloud demonstrates that omitting local context results in substantial computational savings (311.42 s, × 1.79, on the STA dataset) without compromising accuracy. For ${\text{RapidParc}}_{\text{2000}}$ , even a reduction in context_size to 500 (${\text{RapidParc}}_{\text{500}}$ ) during evalutation, still outperforms the baselines while matching the global feature embedding of 500 streamlines in TractCloud more closely (Xue et al., 2023). ${\text{RapidParc}}_{\text{500}}$ requires only 3.15 s for 6.2 million streamlines, over 223 × faster than TractCloud and 124 × faster than GlobalTractCloud. A comprehensive evaluation of RapidParc with different context sizes is shown in the Appendix Figure A.1.

**Table 1.** Quantitative comparison on both the unaugmented test set (200,000 streamlines) and the STA dataset (6.2 million streamlines).

	Test-data – 200,000 streamlines			
	Acc [%]	F1 [%]	Time [s]	CPU-Only [s]
DeepWMA	90.29*	88.12*	n.a.	n.a.
DCNN++	91.26*	89.14*	n.a.	n.a.
TractCloud	92.12	90.22	24.29	271.29
GlobalTractCloud	92.18	90.14	12.67	252.21
RapidParc_500_	93.92 ± 0.06	92.46 ± 0.11	**0.19** ± **0.00**	**4.34** ± **0.02**
RapidParc_2000_	**94.44** ± **0.04**	**93.2** ± **0.09**	0.27 ± 0.00	6.87 ± 0.03
RapidParc_hemiaug_	**94.43** ± **0.04**	**93.18** ± **0.09**	0.26 ± 0.00	6.85 ± 0.02
RapidParc_v8_	**94.45** ± **0.07**	**93.22** ± **0.09**	0.29 ± 0.00	6.87 ± 0.02

	STA-data – 6.2 million streamlines			
	Acc [%]	F1 [%]	Time [s]	CPU-Only [s]
TractCloud	91.69	89.65	703.92	8405.95
GlobalTractCloud	91.71	89.66	392.5	8070.41
RapidParc_500_	92.73 ± 0.06	90.98 ± 0.06	**3.15** ± **0.01**	**130.75** ± **0.28**
RapidParc_2000_	**93.84** ± **0.05**	**92.36** ± **0.08**	5.41 ± 0.00	216.00 ± 2.12
RapidParc_hemiaug_	**93.86** ± **0.02**	**92.36** ± **0.05**	5.44 ± 0.01	215.10 ± 0.37
RapidParc_v8_	**93.82** ± **0.05**	**92.35** ± **0.04**	5.44 ± 0.01	213.56 ± 0.34

Asterisks indicate results reported by Xue et al. (2023) on the identical test set. Subscripts denote the context_size, whether hemisphere dropout (*hemiaug*) was applied, and whether streamlines were downsampled to 8 vertices (*v8*) during training. ${\text{RapidParc}}_{\text{500}}$ was trained on a context_size of 2000 but evaluated on a context_size of 500. Our method achieves state-of-the-art accuracy and F1 scores while being orders of magnitude faster. For RapidParc and RapidParc_hemiaug_, we report mean and standard deviation across five separately trained models. Bold entries mark models whose mean lies within two standard deviations of the column leader.

Comparisons of RapidParc to the registration-based alternatives DeepWMA and DCNN++ illustrate that registration-free parcellation is not only more efficient but also yields higher classification accuracy (Xu et al., 2019; Zhang et al., 2020). Although we were unable to directly compare inference times for DeepWMA and DCNN++ in our environment, prior work has reported that TractCloud is faster than both methods even when registration time is excluded (Xue et al., 2023). During evaluation, RapidParc utilized a maximum of 9.42 GB of GPU memory for context size 2000. Reducing the context_size to 500, matching the global context in TractCloud, further reduces GPU memory consumption to 3.54 GB.

For CPU-only inference, all RapidParc variants processed the 6.2 million-streamline STA dataset in under four minutes on a decade-old 6th-generation Intel i7 CPU, demonstrating robustness and scalability without dedicated hardware acceleration. On in-distribution test data, ${\text{RapidParc}}_{\text{hemiaug}}$ consistently achieved accuracy and F1 scores comparable to RapidParc.

To assess the scalability of RapidParc to large-scale tractograms, we combined two copies of the dTOR dataset (Elias et al., 2024), resulting in 23.64 million streamlines that we classified on a single NVIDIA A40 GPU. Model initialization required 0.5 s. Loading the 24 GB tractogram from disk and pre-processing including subsampling took 394.64 s, while inference on the full dataset completed in 22.46 s. This indicates that computation is predominantly constrained by data I/O and streamline preprocessing rather than model inference.

### 3.2 Performance on external public datasets

**Fig. 3.**
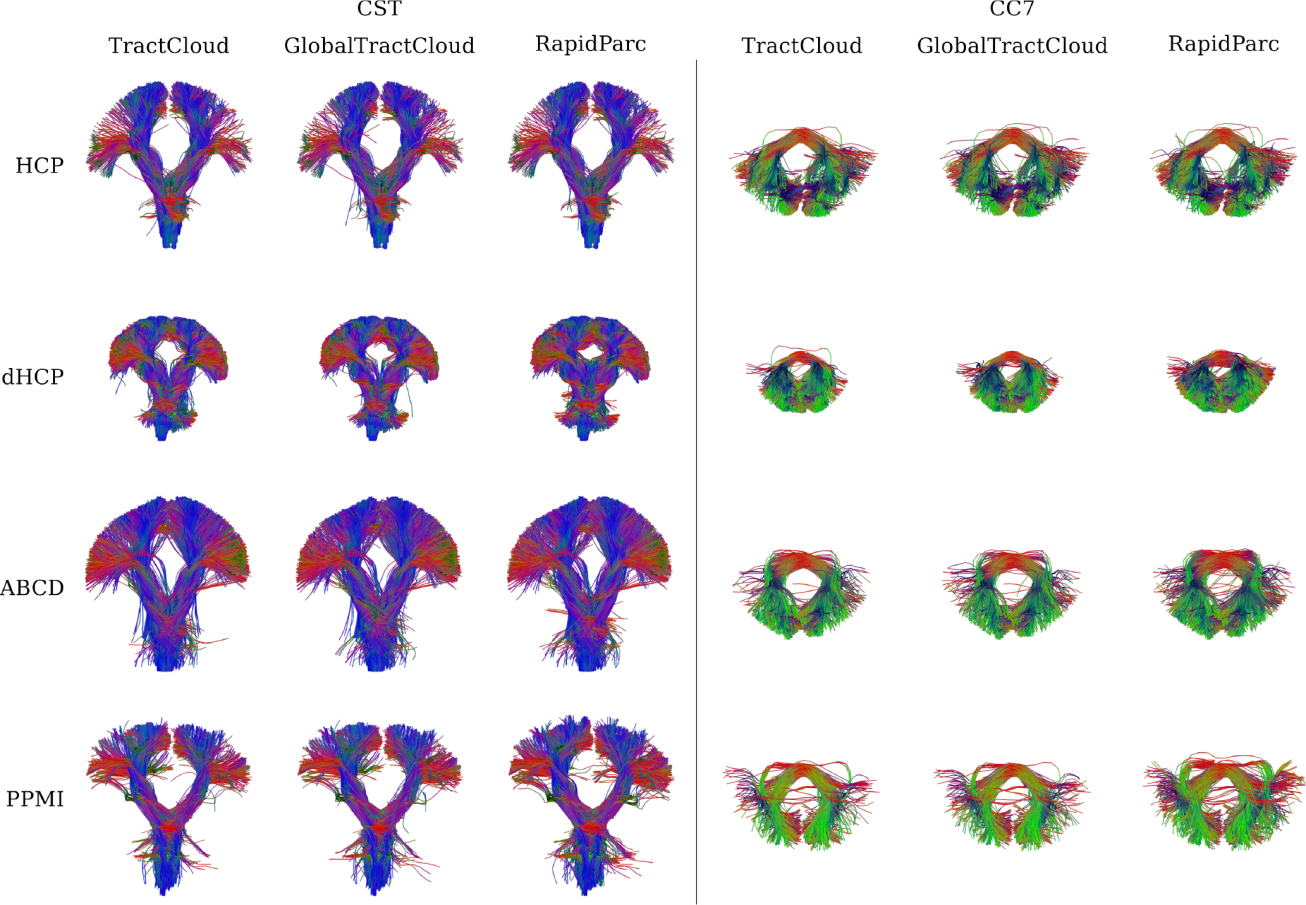
Comparison of RapidParc to TractCloud and GlobalTractCloud on four subjects from different studies. Displayed are parcellation results for the CST and CC7. All models generalize well to different measurement schemes, different ages, and different health status.

To evaluate generalization capability, Figure 3 shows qualitative results on subjects from the ABCD, PPMI, HCP, and dHCP datasets, comparing RapidParc with TractCloud and GlobalTractCloud. All models yielded promising results, including on the challenging dHCP subject. GlobalTractCloud exhibits generalization performance comparable to TractCloud, despite operating without access to local streamline feature context. Overall differences between models are subtle; RapidParc assigns slightly more streamlines to the CC7 compared to TractCloud and GlobalTractCloud.

### 3.3 Performance on subjects post-hemispherotomy and selective amygdalohippocampectomy

To assess model generalizability in the presence of severe structural disruptions, we evaluated parcellation performance in a cohort comprising 22 individuals post-hemispherotomy and 20 demographically matched healthy controls. As a reference, tract assignments were derived using manually guided registration, followed by spectral embedding-based clustering informed by the ORG atlas (O’Donnell et al., 2007; Zhang, Wu, Norton, et al., 2018), allowing for consistent anatomical labeling across subjects. Both qualitative and quantitative analyses were performed to evaluate the accuracy, completeness, and robustness of parcellation results across models, with particular attention to the effects of large-scale white matter disruption in the lesional group.

**Fig. 4.**
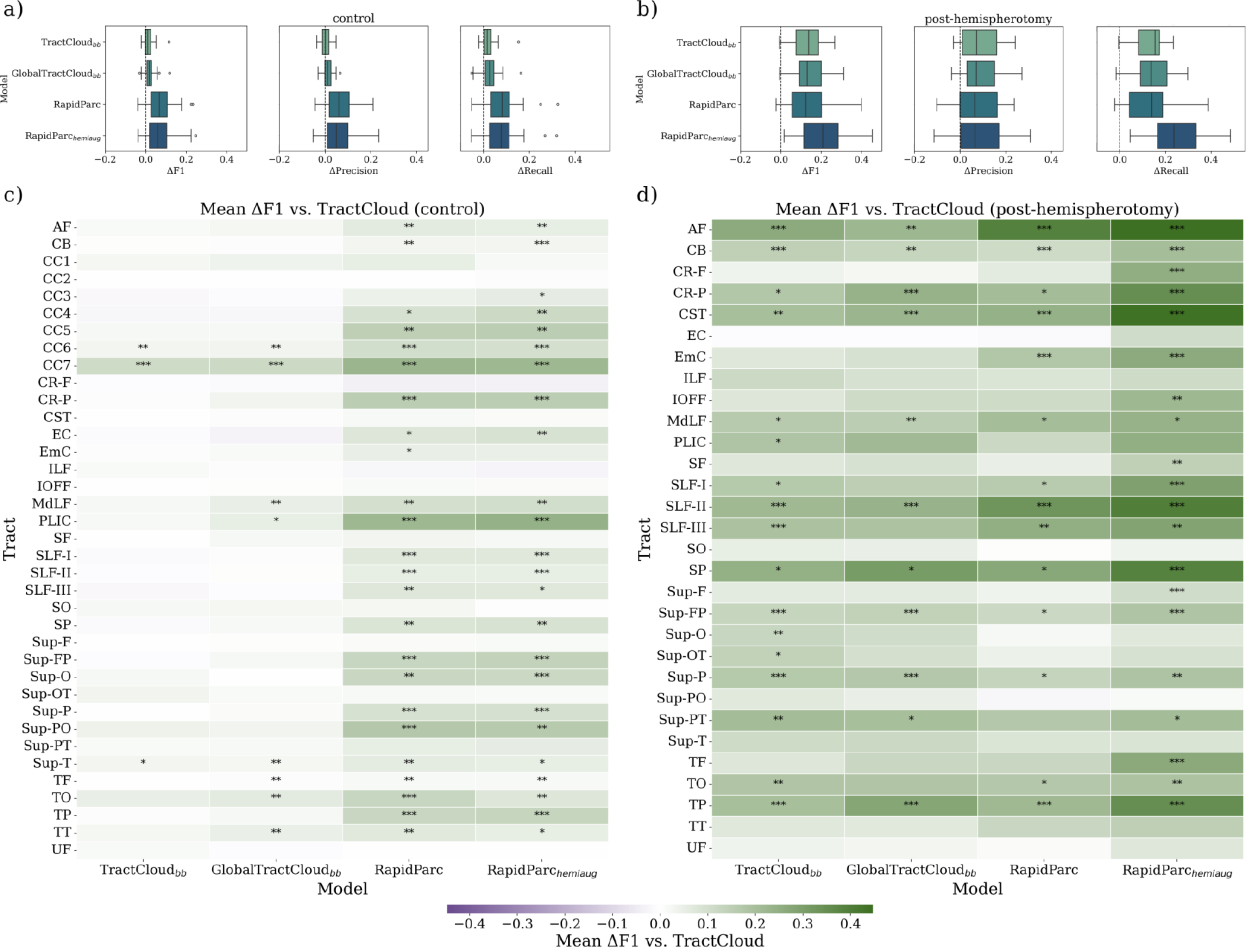
Quantitative comparison of parcellation models on healthy controls and individuals post-hemispherotomy. In (a) and (b), mean change in F1 score, precision, and recall is shown relative to TractCloud, for healthy controls and individuals post-hemispherotomy, respectively. In (c) and (d), Tract-level ΔF1 compared to TractCloud is color coded across individual tracts. Color indicates magnitude and direction of change; asterisks denote statistical significance (*p<0.05 , **p<0.01 , ***p<0.001 ; Bonferroni corrected). Subscript *bb* indicates bounding box centering instead of mean-based centering.

Figure 4 presents a quantitative comparison of TractCloud to its two variants TractCloud_bb_ and GlobalTractCloud_bb_, and to the two variants of our transformer-based approach, RapidParc and ${\text{RapidParc}}_{\text{hemiaug}}$ . Figure 4a and b summarize the overall improvements in F1, precision, and recall, under both control and post-hemispherotomy conditions. Figure 4c and d color-code mean differences in F1 on a per-tract level and indicate significant differences toward TractCloud using asterisks.

Both RapidParc variants outperform the baselines across all metrics, and almost all tracts. In the lesioned cohort, ${\text{RapidParc}}_{\text{hemiaug}}$ exhibits the most pronounced improvements, indicating that hemisphere dropout confers greater robustness to large-scale anatomical disruptions. Improvements are broadly distributed across commissural, association, and projection tracts. Statistically significant differences (Wilcoxon signed-rank test; Bonferroni-corrected across all tests) are observed in a greater number of tracts for both RapidParc variants, with the model augmented with hemisphere dropout showing consistently higher performance in individuals with severe white matter loss.

Since these main findings exclude the outlier class, we additionally monitored false accept and false reject rates. In individuals post-sAH, RapidParc achieves lower false accept (median 8.60% vs. 9.63%) and false reject rates (median 10.53% vs. 11.39%) than TractCloud. In individuals post-hemispherotomy, false reject rates are reduced even more strongly (median 17.45% vs. 21.11%), despite slightly lower false accept rates (median 8.52% vs. 8.86%). This indicates that RapidParc is indeed more robust, not merely more conservative.

**Fig. 5.**
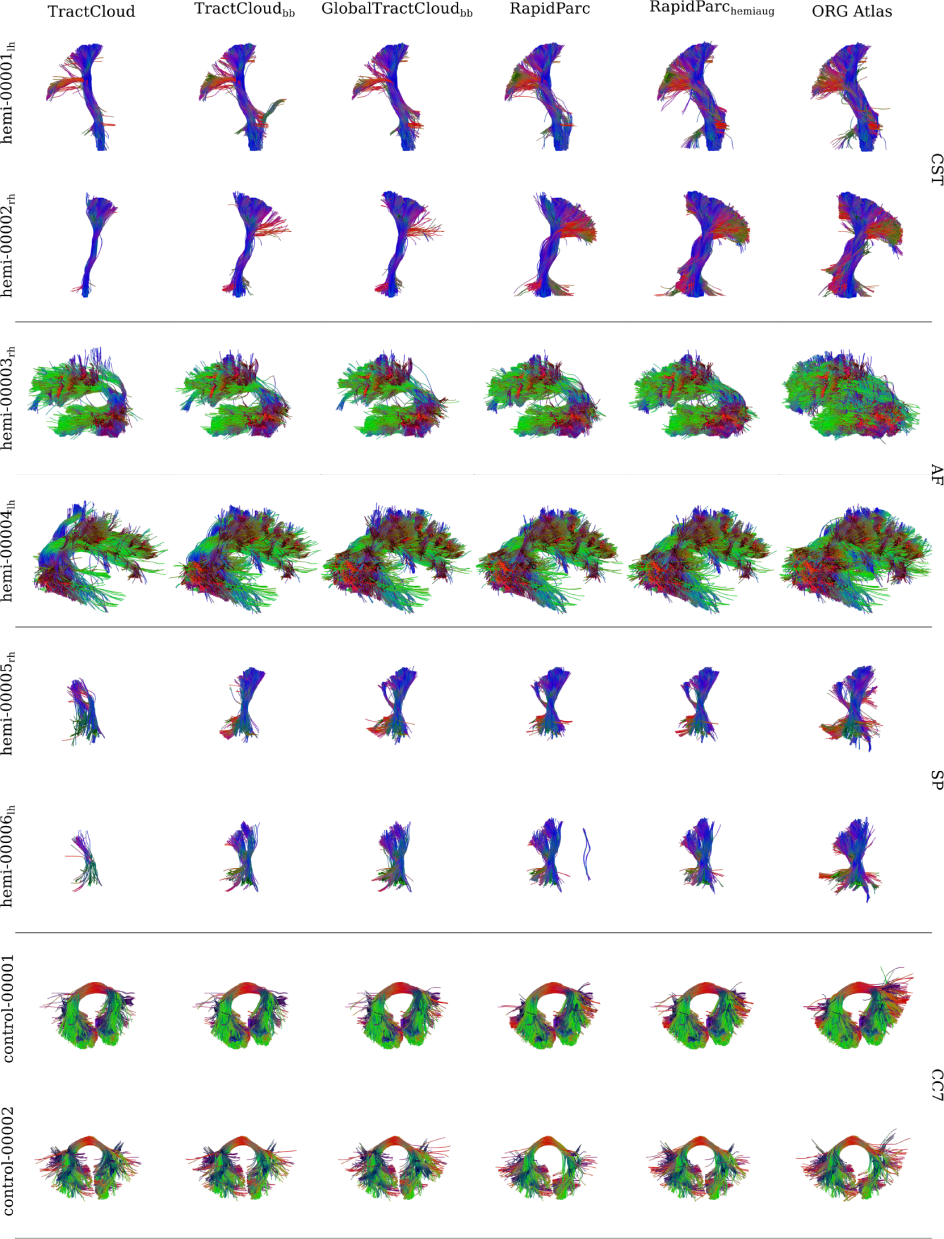
Qualitative assessment of tract parcellation in individuals post-hemispherotomy and healthy controls. One representative example each is presented for TractCloud and RapidParc. The corticospinal tract (CST; rows 1-2), arcuate fasciculus (AF; rows 3-4), and striato-parietal projection tract (SP; rows 5-6) are shown in individuals post-hemispherotomy. In healthy controls (rows 7-8), segment 7 of the corpus callosum (CC7) is displayed. rh and lh refer to the contralesional hemisphere in individuals post-hemispherotomy.

Figure 5 presents a corresponding qualitative assessment, illustrating differences in tract parcellation across models and subject groups. For each group (post-hemispherotomy and healthy controls), white matter tracts with the highest effect size compared to TractCloud are shown, averaged over all models. For each tract, two representative subjects are shown, one for TractCloud and one for RapidParc, based on the respective median F1 scores.

Overall, RapidParc yields more anatomically complete and coherent tract reconstructions compared to TractCloud, which frequently produces fragmented or incomplete bundles in individuals post-hemispherotomy. The use of bounding box-based centering, as implemented in TractCloud_bb_ and GlobalTractCloud_bb_, substantially improves parcellation performance relative to TractCloud’s default mean-based centering, indicating the importance of spatial normalization strategies. Remarkably, GlobalTractCloud_bb_ achieves parcellations that are visually comparable to those of TractCloud_bb_, while requiring significantly fewer computational resources.

The incorporation of hemisphere dropout in RapidParc enhances robustness in subjects post-hemispherotomy, enabling better delineation of affected tracts despite substantial morphological disruption. In healthy controls (Fig. 5, rows 7–8), the effect size of parcellation differences is more subtle, though RapidParc continues to show slightly more complete parcellations. TractCloud with default centering objectively fails to produce viable segmentations for certain tracts, such as the superior parietal (SP) tract in lesional hemispheres (rows 5–6), whereas RapidParc generates anatomically plausible reconstructions.

Table 2 shows that RapidParc variants exceed TractCloud baselines in both accuracy and macro-F1 already in healthy controls. In individuals post-hemispherotomy, this difference further increases, with RapidParc_hemiaug_ showing the strongest gains (72.40% accuracy, 54.17% macro-F1) compared to TractCloud (64.95%, 34.15%). In the cohort post-sAH, RapidParc again dominates TractCloud and its variants. Overall, results indicate improved robustness under different types of white matter disruptions. Notably, TractCloud_bb_ and GlobalTractCloud_bb_ modestly improve over TractCloud, underscoring the impact of more robust centering, but still trail RapidParc. Tract-level macro-F1 ranges reveal substantial heterogeneity across bundles, suggesting that some tracts fail almost completely, while others can be parcellated more robustly.

**Table 2.** Median tract-level macro-F1 (with tract-level range in parentheses) and accuracy medians across groups.

	Healthy controls	
	Acc median [%]	Macro-F1 [%]
TractCloud	73.73	66.26 (24.82–88.28)
TractCloud_bb_	74.31	66.09 (26.87–88.12)
GlobalTractCloud_bb_	74.91	67.08 (30.36–88.42)
RapidParc_2000_	77.45	73.39 (47.29–88.37)
RapidParc_hemiaug_	77.44	73.40 (49.49–89.03)

	Hemispherotomy	
	Acc median [%]	Macro-F1 [%]
TractCloud	64.95	34.15 (9.38–55.79)
TractCloud_bb_	70.54	45.25 (24.10–62.23)
GlobalTractCloud_bb_	70.37	45.94 (25.82–62.27)
RapidParc_2000_	69.87	46.82 (27.56–59.57)
RapidParc_hemiaug_	72.40	54.17 (36.89–69.99)

	sAH	
	Acc median [%]	Macro-F1 [%]
TractCloud	70.94	57.21 (29.12–83.40)
TractCloud_bb_	72.02	60.52 (32.68–84.06)
GlobalTractCloud_bb_	71.77	59.51 (33.64–83.80)
RapidParc_2000_	73.46	63.10 (41.11–86.09)
RapidParc_hemiaug_	73.51	63.53 (39.53–85.70)

Values are reported for healthy controls, individuals post-hemispherotomy, and after sAH.

**Fig. 6.**
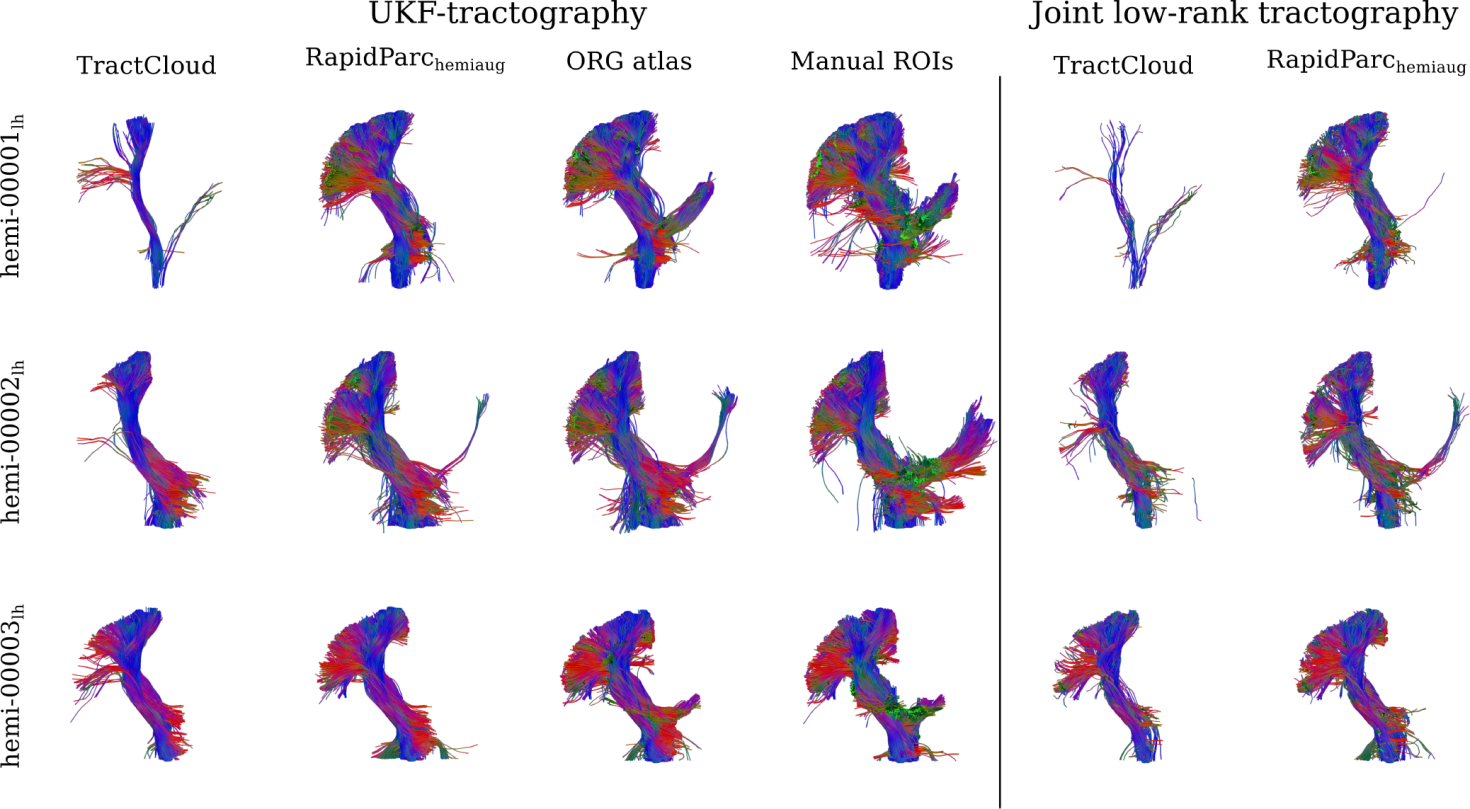
Corticospinal tract parcellations for three individuals post-hemispherotomy. Columns show TractCloud, RapidParc, the ORG-atlas-based result, and manual ROI dissections. Residual lesional fibres appear inconsistently across automated methods, underscoring the case-dependent difficulty of recovering truncated bundles. In the right two columns, parcellations of the same subjects are shown, obtained from tractograms generated with probabilistic joint low-rank tractography (Gruen et al., 2023) rather than UKF-tractography.

A noteworthy observation in our experiments was the way in which the models deal with remnants from surgically disconnected tracts, as illustrated in Figure 6. These residual fragments differ from all streamlines in the training set. Therefore, none of the available methods handle them in a clearly predictable fashion. This remained true even when trying out another tractography algorithm, as shown in the right two columns of Figure 6.

## 4 Discussion

Registration-free tractogram parcellation is particularly advantageous for populations with extensive white matter disruption, where automated registration often fails or produces distorted alignments, while manually guided registration is time consuming and less reproducible. RapidParc, our proposed novel method, provides accurate parcellations across datasets from diverse sources, including pediatric and neurodegenerative populations, as well as patients with large-scale lesions, without requiring dataset-specific retraining or manual intervention.

In particular, we are the first to evaluate registration-free parcellation on a challenging cohort of patients after hemispherotomy. RapidParc produces more complete reconstructions in these cases compared to TractCloud, the previous state-of-the-art registration-free method. In addition, RapidParc is up to two orders of magnitude faster, and slightly more accurate even on in-distribution data.

Our ablation studies indicate that the observed improvements in lesion robustness are due to two main factors: First, our method is trained with powerful data augmentation. In particular, we newly introduce the idea of hemisphere dropout, which encourages the model to focus on context from the ipsilateral hemisphere and thereby limits the effects of large-scale anatomical disruptions on the opposite hemisphere. This helps to retain the ability to accurately segment surviving tracts, even when prior methods fail to produce anatomically complete parcellations. Future work might explore further patient cohorts and augmentations, such as deformations based on models of tumor growth (Young et al., 2022).

Second, we perform spatial normalization differently than the previous state of the art, using the bounding box of the overall tractogram rather than its center of mass, which can be biased by asymmetric streamline density, shifting the tractogram centroid away from the true anatomical midline. We demonstrate that augmentations in TractCloud are insufficient to deal with severe unilateral tract loss, and that replacing its centering strategy with one similar to ours improves recall in such cases. Even though this represents a clear advancement, future work might explore even more robust methods for centering, based on a dedicated model for estimating the anatomical center from a tractogram or, when additional imaging data is available, a skull mask.

On the other hand, our experiments indicate that the local part of the local-global context used in TractCloud does not make a clear contribution to its accuracy, while imposing significant computational overhead. The fact that our architecture leverages a purely global context is a key reason for its computational efficiency, enabling a high level of parallel processing and eliminating the need for costly per-streamline neighborhood construction. Future work might explore dedicated embedding strategies such as TractoEmbed (Goel et al., 2024) which include spatial information about regional patches, neighborhoods, and the streamline itself, while still enabling fully parallel processing.

In the present study, we had to exclude the cerebellar tracts from our analysis of patients after hemispherotomy, due to the reduced field-of-view in the respective dMRI acquisition. Recent work by Chen, Zekelman, et al. (2025) demonstrated that augmenting tractograms by cutting off cerebellar streamlines can improve generalization on tractography data with reduced FOVs. In the future, we plan to explore whether including similar strategies in RapidParc might further enhance model robustness.

Finally, we observed that lesional fragments, such as the ones in Figure 6, are not handled systematically by any of the currently available learning-based approaches, due to their dissimilarity to the training data. We believe that, based on such streamlines alone, it is impossible to decide with certainty whether they terminated prematurely due to a tracking error, and should therefore be discarded, or whether they represent anatomically plausible fragments, which might be of substantial interest in assessing cases after surgery, stroke, or shear injuries (Jang, 2020; Nolan et al., 2020). While future work could truncate tracts as an additional data augmentation, we therefore propose that such tracts should be assigned to a separate class for manual inspection. Similarly, given suitable training data, U-shaped fibers and short-range association tracts, (Kirilina et al., 2020) which are currently not included in the ORG atlas or in the RapidParc parcellations, could also be included as a separate class.

## 5 Conclusion

RapidParc is a novel, transformer-based model for fast, accurate, and lesion-robust tractogram parcellation that relies solely on global context. It surpasses prior state-of-the-art methods in accuracy and macro F1 score while achieving up to 223 × faster inference through fully parallel tractogram processing. Our findings highlight the scalability and robustness of global context-based parcellation for large-scale and lesioned datasets, and demonstrate the potential of anatomically informed augmentation strategies to improve model generalization in cases with severe white matter disruptions.

## Data Availability

The code and pretrained models are available from https://github.com/MedVisBonn/RapidParc. The training data used in this study is available from the TractCloud repository (Xue et al., 2023). The hemispherotomy data were acquired at the University Hospital Bonn, and are available from the corresponding author upon reasonable request. The data from the ABCD, PPMI, HCP, and dHCP studies are available from their respective repositories (Casey et al., 2018; Edwards and et al., 2022; Marek et al., 2018; Van Essen et al., 2012; Volkow et al., 2018).
